# Effect of iodine nutritional status on the recurrence of hyperthyroidism and antithyroid drug efficacy in adult patients with Graves’ disease: a systemic review

**DOI:** 10.3389/fendo.2023.1234918

**Published:** 2023-10-11

**Authors:** Qingxing Xie, Xinyi Zhang, Jinfang Ma, Xi Lu, Yuwei Zhang, Nanwei Tong

**Affiliations:** Department of Endocrinology and Metabolism, Center for Diabetes and Metabolism Research, Laboratory of Diabetes and Islet Transplantation Research, West China Hospital of Sichuan University, Chengdu, China

**Keywords:** Graves’ disease, hyperthyroidism, antithyroid drug, iodine status, recurrence

## Abstract

**Aim:**

To probe the appropriate iodine nutritional status for patients with Graves’disease (GD) hyperthyroidism and on antithyroid drugs (ATD) or after drugwithdrawal.

**Method:**

Studies were retrieved from three databases (Embase, Medline, and Cochrane Library) and were screened and evaluated using predefined criteria. The risk of bias of each trial was assessed using a tool from Cochrane. The iodine nutritional status of the subjects was redefined according to the World Health Organization (WHO) criteria and classified as insufficient/adequate/above requirements/excessive iodine intake.

**Result:**

Two randomized controlled trials (RCTs) and 3 observational studies were selected from the 376 retrieved papers, which had different degrees of risk of bias in study design. The heterogeneity among them prevented us from further synthesizing effect indicators and subsequent statistical analyses. Two RCTs with high quality showed that insufficient or above requirements iodine intake was detrimental for ATD-treated GD patients; adequate iodine intake was associated with a lower risk of recurrence and better efficacy in controlling thyrotoxicosis. It could be speculated from three low-quality observational studies that excessive iodine intake may be associated with higher (or similar) recurrence rates and lower remission rates compared to above requirements iodine intake in these patients, but none of them could answer the question of the effect of insufficient or adequate iodine intake on this issue.

**Conclusion:**

Although the available evidence is suboptimal, this systematic review tentatively suggests that in adult patients with GD hyperthyroidism receiving ATDs and according to WHO criteria for iodine nutritional status, adequate iodine intake is associated with a lower recurrence rate, a higher remission rate and a better efficacy to control thyrotoxicosis than insufficient, above requirement, or excessive iodine intake. Future RCTs with large samples are expected to elucidate the actual impact of different iodine nutritional statuses on the recurrence rate of hyperthyroidism and the efficacy of ATD to control thyrotoxicosis in these patients.

**Systematic review registration:**

identifier CRD42022359451.

## 1 Introduction

In recent years, salt iodization projects have been implemented worldwide under the call of the World Health Organization (WHO), making iodine deficiency effectively controlled (1), along with an increasing proportion of people with appropriate iodine nutritional status. Hyperthyroidism is a major endocrine disorder, of which Graves’ disease (GD) is the most common cause. Iodine, an important raw material for the synthesis of thyroid hormones, is involved in its pathogenesis (2). Concerns about the relationship between iodine intake and autoimmune thyroid diseases, including GD, have emerged as the iodine nutritional status of the population has improved (3, 4).

Opinions about the relationship between the incidence or prevalence of thyroid disease and iodine intake vary around the world (5–10). Among patients with GD hyperthyroidism and on antithyroid drug (ATD) therapy, studies exploring the effect of dietary iodine intake (i.e., iodine nutritional status) on the recurrence of hyperthyroidism are scarce and with small sample sizes. To make it worse, inconsistent definitions of iodine nutritional status of different research have led to confusion and misunderstanding. In clinical practice, endocrinologists guided by traditional views often recommend a low iodine diet for patients with GD hyperthyroidism to maintain their iodine deficiency status (11); our patients are also confused as to whether they should go for iodized or non-iodized salt and whether their iodine intake should be severely restricted. Due to the lack of support from evidence-based medicine, the latest international guidelines for the management of GD hyperthyroidism seem to avoid discussion on this issue (12–14), while clinicians must answer the question of iodine intake from their patients in clinical practice.

Therefore, there is an urgent need to redefine the reported iodine nutritional status using uniform criteria to avoid confusion due to various descriptions from different authors. To this end, we have completed a systematic review to evaluate the effect of different iodine nutritional statuses on the recurrence of GD hyperthyroidism after ATD treatment using a uniform redefinition according to WHO criteria. The study will provide evidence-based conclusions on what iodine nutritional status should be maintained during the treatment of GD hyperthyroidism and guide clinicians in the management of iodine intake in these patients.

## 2 Method

### 2.1 Literature review methodology and results

We searched all the literature (to 9 September 2022) in the Medline (Ovid SP), Embase (Ovid SP) and Cochrane databases, and there was no language restriction. The 3 involved key words included “graves’ disease”, “antithyroid agents”, and “iodine”; the detailed search strategy is accessible in **Supplementary Material 1**.

### 2.2 Inclusion and exclusion criteria for clinical intervention studies

Inclusion criteria: 1) it was an RCT, cohort study, or case−control study, 2) the disease studied was GD hyperthyroidism, 3) the study was conducted in patients with GD hyperthyroidism on ATD or after drug withdrawal, 4) there were quantitative indicators describing iodine nutritional status, 5) the outcome related to recurrence or remission of hyperthyroidism, and 6) participants had received ATD for at least 6 months and were followed up for at least 6 months after drug withdrawal.

Exclusion criteria: 1) the study did not aim to explore the influence of dietary iodine on the recurrence of GD hyperthyroidism or the efficacy of ATDs, and 2) the study population included pregnant or lactating women or adolescents.

### 2.3 Criteria in the analysis and evaluation of the study

1. Diagnosis of GD hyperthyroidism: GD is diagnosed by composite indicators including clinical manifestations of thyrotoxicosis, elevated thyroid hormone, decreased thyroid stimulating hormone (TSH), and elevated thyrotropin receptor antibody (TRAb) and/or ultrasound suggesting abundant thyroid blood flow (13). Clinical diagnosis of GD is made after excluding other causes of persistent thyrotoxicosis.
2. Recurrence of hyperthyroidism: ATD treatment for more than 6 months to achieve remission criteria (remission of thyrotoxic symptoms and normalization of thyroid hormone), followed by reappearance of clinical manifestations of thyrotoxicosis, elevated thyroid hormone and decreased TSH after drug withdrawal (12–14).
3. Criteria for iodine nutritional status: according to the urinary iodine concentration (UIC) criteria from WHO: a UIC level < 100 μg/L indicates insufficient iodine intake; a level between 100 and 199 is adequate iodine intake; a level between 200 and 299 μg/L is above requirements iodine intake, and ≥ 300 μg/L indicates excessive iodine intake (15).

### 2.4 Clinical intervention studies included in the analysis

**Figure 1:**
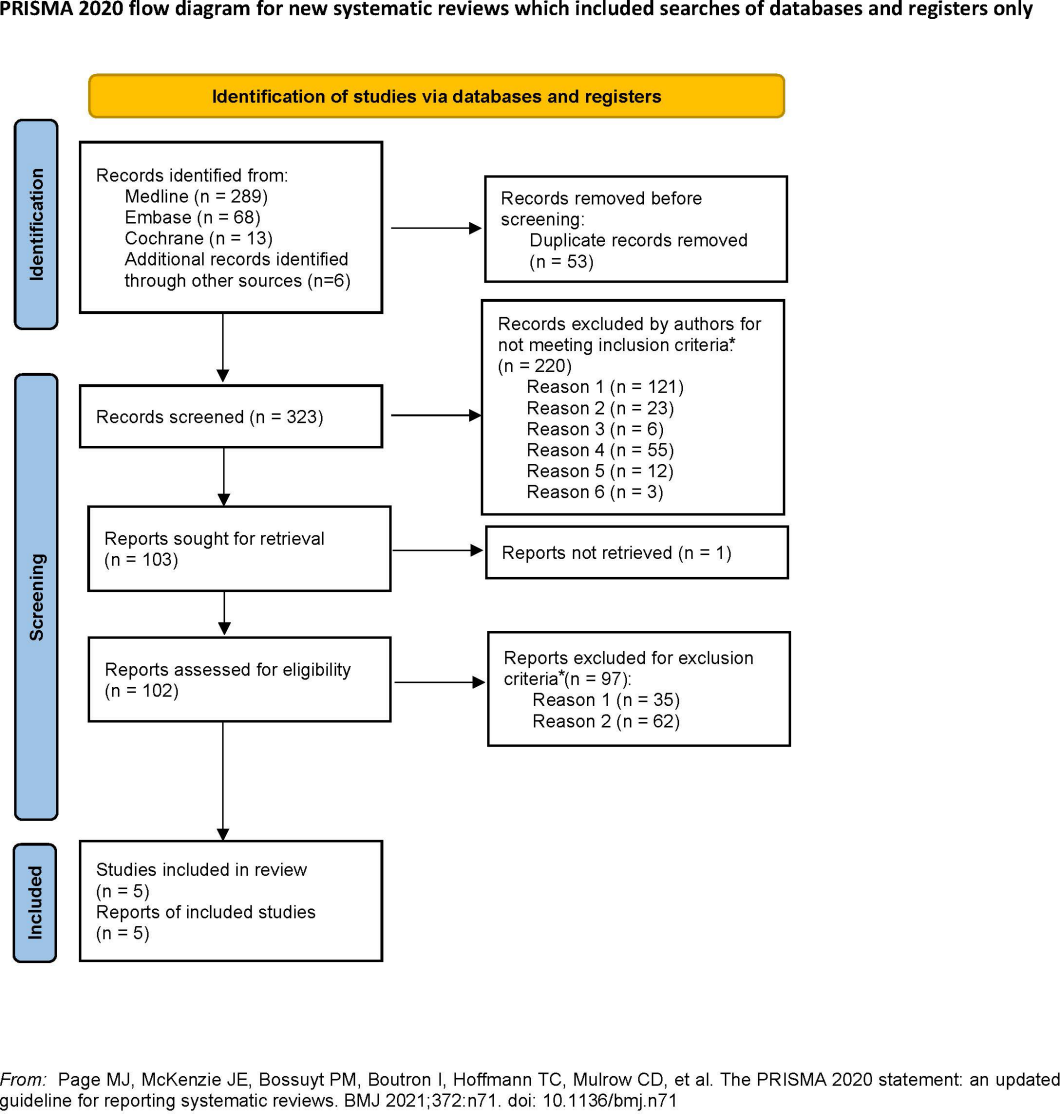
PRISMA flow diagram showing the systemic review process.

Two authors carried out the course of screening and data collection independently. Of the 376 articles retrieved, two RCTs and three observational studies were finally included in the analysis after removing 53 duplicates, reading the titles, abstracts, and full texts, and screening using the preset inclusion and exclusion criteria (**Figure 1**).

### 2.5 Data collection

We extracted information about the year and country in which the trial occurred, the participants’ characteristics, the regime and cases of intervention and control, the evaluating method, the follow-up time, and the study design from each study. We classified the trials into two categories: 1) RCTs with outcomes about recurrence or efficacy and 2) observational studies with outcomes about recurrence.

However, given the limited trials that met our criteria and differences in the study design, outcome indicators and follow-up time, the heterogeneity became nonnegligible and stopped us from synthesizing the outcome, thus we used the original data instead.

### 2.6 Study evaluation and bias analysis

The purpose of this study was to evaluate the effect of different iodine nutritional statuses on the recurrence of hyperthyroidism in adult patients with GD and on ATDs. Accordingly, we set a series of evaluation criteria: 1) to uniformly rejudge the iodine nutritional status according to WHO criteria and based on the authors’ descriptions, 2) to evaluate whether the definition for diagnosis, recurrence and remission of GD hyperthyroidism specified in each study met our preset criteria, and 3) to evaluate whether the duration of ATD treatment and the follow-up period after drug withdrawal met our preset standards.

Two authors worked together to evaluate the bias of each study using version 2 of the Cochrane tool for assessing risk of bias in randomized trial (RoB2) and the risk of bias in nonrandomized studies-of exposure (ROBINS-E) tool.

## 3 Results

### 3.1 RCTs

The information of the two eligible RCTs is listed in **Table 1** and described separately as follows.

**Table 1:** Information extracted from RCTs.

Article information			Country	Participants		Intervention	Control	Intervention period	Outcome	Reference
Author	Year	Journal		Characteristic	Age					
Huang, H.	2018	Clinical Endocrinology	China	Patients newly diagnosed with GD^a^ (randomly assigned after ATD^b^ therapy and dietary iodine restriction for 1 month) (N = 459)	16-65 years old	Iodine-supplemented group: 10 grams of iodized salt/ day (UIC^c^ about 155μg/L) (N = 203)	Iodine-restricted group: nonionized salt with low-iodine or noniodine diet (UIC about 35μg/L) (N = 202)	24 months (ATD+I^d^ for 12m, I for 12 m)	The overall relapse rate within 12 months after ATD withdrawal was significantly higher in the iodine-restricted group (insufficient iodine intake) than in the iodine-supplemented group (adequate iodine intake) (45.5% *vs.* 35.5%, HR = 1.381, *P* = 0.04)	(16)
Dai, W. X.	2006	Chin Med J (Engl)	China	Patients with untreated GD (given USI^i^ before treatment) (N = 124)	14-65 years old	Group A: non-iodinated salt (UIC about 148μg/L) (N = 45)	Group B: USI (35±15mg iodide/kg) (UIC about 242.8μg/L) (N = 56)	6 months (ATD+I)	The PTU dose required to maintain normal thyroid function within 6 months was significantly higher in group B (above requirements iodine intake) than in the group A (adequate iodine intake) (*P* < 0.001)	(17)

^a.^GD, Graves’ disease; ^b.^ATD, antithyroid drug; ^c.^UIC, urinary iodine concentration; ^d.^I, intervention or control.

#### 3.1.1 RCTs with outcomes about relapse

In 2018, an RCT from Fujian, China, examined the relationship between iodine nutritional status and recurrence rate in ATD-treated GD hyperthyroidism patients. After one month of ATD treatment with strict iodine restriction, 459 patients with newly diagnosed GD hyperthyroidism were randomly assigned to the iodine supplementation group (10 g iodized salt, approximately 200 μg iodine/day, n = 203) and the iodine restriction group (non-iodized salt + low iodine diet, n = 202). ATD treatment was withdrawn after 12 months, and follow-up was continued for 12 months (24 months in total), with fasting UIC measured every 3 months and the recurrence rate observed.

There was no significant difference in baseline UIC between the two groups (approximately 60 μg/L), that is, insufficient iodine intake by WHO criteria. After starting the dietary iodine intervention, the UIC was maintained at 155 μg/L (adequate iodine intake by WHO criteria) in the iodine supplementation group and 35 μg/L (insufficient iodine intake by WHO criteria) in the iodine restriction group. After 9 months of intervention, the TRAb in the insufficient iodine intake (iodine restriction) group began to be significantly higher than that in the adequate iodine intake (iodine supplementation) group (*P* < 0.001), which lasted until the end of follow-up (24 months). The overall recurrence rate was significantly higher in the insufficient iodine intake group than in the adequate iodine intake group within 12 months after ATD withdrawal (45.5% *vs.* 35.5%, HR = 1.381, *P* = 0.04) (16).

In this trial, after the dietary iodine intervention, the actual iodine nutritional status of the two groups was insufficient iodine intake and adequate iodine intake. During ATD treatment, although there was no significant difference in FT4 and TSH between the two groups, TRAb was significantly higher in the insufficient iodine intake group, suggesting a high risk of hyperthyroidism recurrence. The difference in the recurrence rate of hyperthyroidism after ATD withdrawal further demonstrated the relationship between insufficient iodine intake and a high recurrence rate. Compared with insufficient iodine intake, adequate iodine intake reduces the risk of recurrence of GD hyperthyroidism after ATD withdrawal.

The definition of hyperthyroidism recurrence and the duration of ATD treatment in this study were in accordance with guideline recommendations. The UIC was measured every 3 months, which not only allowed for a dynamic assessment of the participants’ iodine status but also suggested that the dietary intervention could successfully influence the iodine nutritional status. This trial showed a lower risk of recurrence of hyperthyroidism in ATD-treated GD patients with adequate iodine intake (compared to insufficient iodine intake) but lacked evidence about the above requirements or excessive iodine intake.

#### 3.1.2 RCTs with outcomes about efficacy

In 2006, an RCT in Beijing, China, included 124 patients with newly diagnosed GD, all using universal salt iodization (USI) and without dietary iodine restriction prior to treatment. Patients were randomized into two groups: group A was given non-iodized salt (n= 45), and group B was given USI (35 ± 15) mg iodide/kg (n = 56). The follow-up period was 6 months after the beginning of ATD treatment, with UIC measured once before ATD treatment and once approximately 3 months after the start of treatment.

The median baseline UIC for participants in both groups was approximately 220 μg/L (above requirements iodine intake by WHO criteria). The dietary iodine intervention led to a median UIC of 148.4 μg/L (adequate iodine intake by WHO criteria) in the iodine restriction group and 242.8 μg/L (above requirements iodine intake by WHO criteria) in the USI group. Within 6 months after the beginning of ATD treatment, there was no significant difference in FT4 level and TRAb positivity between the two groups. However, from the second month onward, the ATD dose required to maintain normal thyroid function was significantly higher in the above requirements iodine intake group (using USI) than in the adequate iodine intake group (iodine restriction) (*P* < 0.001). The difference persisted until the end of the follow-up (17).

Although the trial differed from Fujian 2018 in terms of follow-up time and outcome indicators (lacking evaluation regarding recurrence rates), it was found that the ATD dose required to maintain normal thyroid function was significantly lower in the adequate iodine intake group. Since the dose of ATD for maintenance of thyroid function was associated with recurrence of GD hyperthyroidism, we hypothesized that the risk of recurrence after ATD withdrawal in the adequate iodine intake group was lower compared with the above requirements group. In addition, it was found that TRAb, an indicator associated with the risk of recurrence of GD hyperthyroidism, showed a decreasing trend in the adequate iodine intake group, while no such trend was observed in the above requirement iodine intake group. Perhaps the difference would have been more pronounced if the duration of intervention and follow-up had been extended to 12 months [as recommended by a meta-analysis on the reasonable duration of ATD treatment for GD (18)] with larger sample size.

The authors originally concluded that iodine intake should be restricted in patients with GD, but after correcting for iodine nutritional status by WHO criteria, their restricted iodine intake group was actually with adequate iodine status. Thus, we think it seems more appropriate to conclude that “adequate iodine intake may be associated with a better effect of ATDs and a lower risk of recurrence compared with iodine above normal”. The study measured UIC once before and after starting the intervention, indicating that dietary iodine restriction did not result in insufficient iodine intake in participants. The outcome indicator was the dose of ATD needed to maintain normal thyroid function within 6 months after starting treatment, but there was a lack of evidence on the recurrence rate of hyperthyroidism after drug withdrawal. In addition, the treatment course is short.

Although baseline and postintervention UIC level, ATD intervention time and outcome indicators were inconsistent between the two RCTs, the effect of iodine nutritional status on the recurrence of hyperthyroidism in ATD-treated GD patients tended to be the same. It can be concluded that insufficient or above required iodine intake is detrimental to ATD-treated GD patients; adequate iodine intake is associated with a lower risk of recurrence and a better efficacy to control thyrotoxicosis.

### 3.2 Observational studies with outcome about relapse

A cohort study from Korea in 2015 included patients who had taken ATD for at least 12 months and withdrawn drugs after achieving euthyroid function. They were followed for more than 12 months (a median of 23 months) to observe the presence of recurrence, and UIC was measured before and after ATD withdrawal. There were no significant differences in the mean UIC (412 μg/l *vs*. 408 μg/l, both excessive iodine intake) and UI/Cr (309 *vs*. 320 μg/g) between the remission and recurrence groups. Then, according to the Korean per capita UIC (358 μg/l, excessive iodine intake by WHO criteria), patients with UIC <300 μg/l (non-excessive iodine intake by WHO criteria) were defined as the average iodine intake group (n = 52), and patients with UIC ≥300 μg/l (excessive iodine intake by WHO criteria) were defined as the excess iodine intake group (n = 90), with the latter having a higher recurrence rate than the former, but no significant difference (30% *vs*. 21.2%, *P* = 0.20) (19). The authors concluded that in areas with high levels of iodine intake, it may not be necessary to restrict dietary iodine intake in ATD-treated GD patients. The definition of hyperthyroidism recurrence and the duration of ATD treatment in this trial were in accordance with guideline recommendations. UIC was tested during treatment and after drug withdrawal (without the exact time of testing), but the iodine status was estimated to be the same before and after treatment. The mean UIC of the participants during ATD treatment and after drug withdrawal was redefined as non-excessive iodine intake (the mean UIC was 358 μg/l, while the UIC of this group was <300 μg/l, so it was presumed to belong mainly to the above requirements iodine intake) and the excessive iodine intake group according to WHO criteria. Therefore, it can only be concluded that the recurrence rate of hyperthyroidism in patients with GD after ATD withdrawal is the same when comparing the above iodine intake requirements with the excessive iodine intake. However, the effect of insufficient or adequate iodine intake compared with other iodine nutritional statuses on recurrence cannot be answered.

In 1987, a case−control study in the United States included 106 patients with GD hyperthyroidism treated with ATD for at least 6 months. Remission was defined as no relapse for at least 6 months after ATD withdrawal. From 1973 to 1985, the remission rate increased gradually (r = 0.60, *P* < 0.02), while the estimated daily iodine intake decreased (r = -0.81, *P* < 0.009) (20). Although the trial did not offer information about UIC, it provided data on daily iodine intake. We found that an estimated daily iodine intake between 200-300 μg/day (estimated above requirements iodine intake by WHO criteria) corresponded to a higher remission rate, when comparing with that between 700-800 μg/day (estimated excessive iodine intake by WHO criteria). In this study, the subjects received a short course of ATD, and the UIC was not measured, nor was the method of estimated daily iodine intake specified. Therefore, it was not possible to accurately assess the subjects’ iodine nutritional status. As far as iodine intake is concerned, the WHO recommendation for adequate daily iodine intake for adults is 150 μg (21), so the subjects’ iodine intake was still higher than WHO recommendation. The study did not report an explicit recurrence rate, but its definition of remission suggests its association with recurrence, so it is speculated from the data given in the study that excessive iodine intake may be associated with a lower rate of remission of hyperthyroidism (and presumably a higher rate of recurrence) for ATD-treated GD patients, but the effect of different iodine nutritional statuses on hyperthyroidism recurrence and remission cannot be answered.

In 1965, a historical control study in Britain compared the recurrence rate within 6 months after drug withdrawal in 16 GD hyperthyroidism patients who were supplemented with 200 μg potassium iodide daily (in 1965) and 41 patients who were not supplemented with iodine (in 1964). Plasma inorganic iodine (PII) was measured after ATD withdrawal. The authors concluded that the recurrence rate was higher in the iodine-supplemented group than in the noniodine-supplemented group (56% *vs*. 27%, *P* < 0.05) (22). However, there are no studies on the correspondence between PII and iodine nutritional status evaluated with UIC, preventing us from assessing them by WHO criteria. According to a previous report by this author, normal PII values of all ages were 0.04-0.57 μg/100 ml (23). The noniodine supplemented group were historical controls from the previous year, and a significant decrease in their PII was observed within 90 days after ATD withdrawal (from 0.21 ± 0.052 μg/100 ml to 0.04 ± 0.012 μg/100 ml, but still within the normal rage specified by the authors and lack of specific iodine nutritional status by WHO criteria). The iodine supplemented group, which was given 200 μg potassium iodide/day immediately after drug withdrawal, had a PII of 0.27 μg/100 ml both at the time of withdrawal and 6 months after that. Combined with the normal range given by the authors, the iodine supplemented group might not be in a state of iodine deficiency, indicating iodine overdose by daily supplementation of potassium iodide. Therefore, the high recurrence rate in this trial was actually associated with estimated excessive iodine intake (lack of specific iodine nutritional status by WHO criteria). The study did not provide a clear definition of hyperthyroidism recurrence and ATD treatment duration, nor did it measure UIC, leading to confusion about iodine nutritional status. Moreover, the study was a historical control study with confounding factors such as time span, patient compliance, and ATD treatment duration that have important effects on recurrence, so no conclusions could be drawn about the effects of different iodine statuses on the recurrence rate of GD hyperthyroidism after ATD treatment.

It was speculated from the results of the three observational studies that excessive iodine intake may be associated with high or similar recurrence and low remission rates comparing with above requirements iodine intake in ATD-treated GD patients. However, none could answer the effect of insufficient or adequate iodine intake on that issue (**Table 2**).

**Table 2:** Information extracted from observational studies.

Article information			Country	Participants Characteristic	Study design (group)	Follow-up Duration	Outcome	Reference
Author	Year	Journal						
Park, S. M.	2015	European Thyroid Journal	Korea	Patients with GD^a^ who had taken ATD^b^ for at least 12 months to achieve euthyroid function and then stopped their ATD (N = 142)	Average iodine intake group (group 1): UIC^c^< 300 μg/L (N = 52); excessive iodine intake group (group 2): UIC ≥ 300 μg/L (N = 90)	23 months on average	No significant difference happened in the relapse rate between group 2 (excessive iodine status) and group 1 (average iodine status) about 2 years after ATD withdrawal (30% *vs.* 21.2%, *P* = 0.20)	(17)
Solomon, B. L.	1987	Annals of Internal Medicine	USA	Patients with an established diagnosis of GD (N = 69)	Investigate the remission rate and estimated iodine intake (from 700-800 μg/day to 200-300 μg/day) respectively in 1973-1985	At least 6 months	The remission rate gradually increased with year (from less than 10% to over 30%) (r = 0.60, *P* < 0.02), while the estimated daily iodine intake decreased (r = -0.81, *P* < 0.009)	(18)
Alexander, W. D.	1965	Lancet	Britain	Patients with GD after withdrawing ATD (N = 57)	Intervention group (group 1, 1965): received iodide supplements with normal PII^d^ (N = 16); Historical control (group 2, 1964): did not receive any iodide supplements with low PII (N = 41)	At least 6 months	The relapse rate was higher in the group 1 (estimated excessive iodine status) than in the group 2 (estimated iodine deficiency) (56% *vs.* 27%, *P* < 0.05)	(20)

^a.^GD, Graves’ disease; ^b.^ATD, antithyroid drug; ^c.^UIC, urinary iodine concentration, ^d.^PII, plasma inorganic iodine.

**Figure 2:**
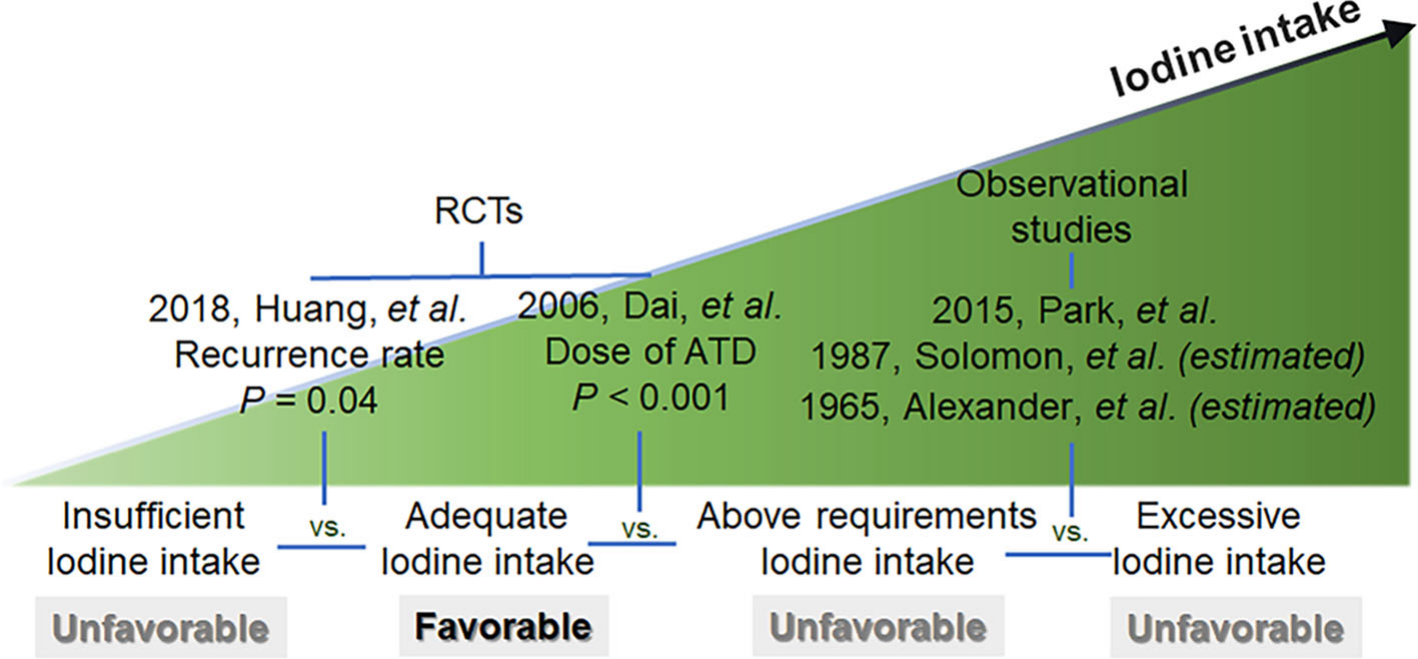
Summary Chart for trials.

Integrating all the above findings and inferences, we conclude that the recurrence rate of hyperthyroidism is lower and the remission rate is higher with adequate iodine intake compared to insufficient or above requirements or excessive iodine intake in ATD-treated GD patients (**Figure 2**).

### 3.3 Risk of bias

**Figure 3:**
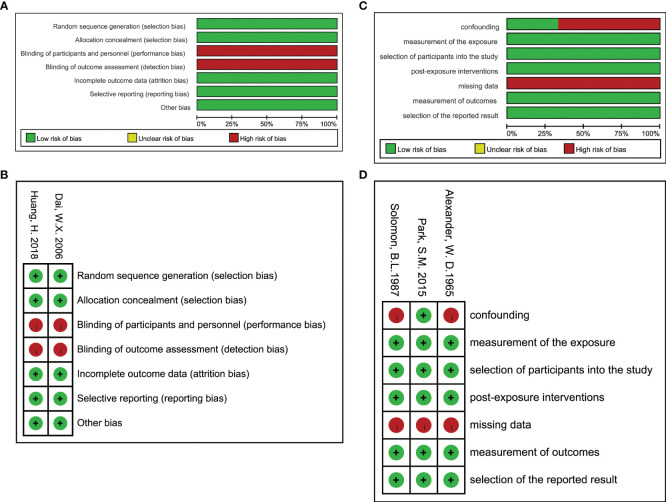
Risk of bias (using ROB2 for RCTs and ROBINS-E for observational study). **(A)** Risk of bias graph for RCTs: authors’ judgements about each risk of bias item presented as percentages across all included RCTs. **(B)** Risk of bias summary for RCTs: authors’ judgements about each risk of bias item for each included RCTs. **(C)** Risk of bias graph for observational studies. **(D)** Risk of bias summary for observational studies.

The risk of bias assessments for each study are summarized in **Figure 3**.

Both RCTs were not double-blinded, so there were some concerns regarding the measurement of the outcome, but the outcome indicators (thyroid hormone, TRAb, and UIC) were objective quantitative indicators with little subjective influence from the participants, so the risk of bias in this area was considered to be low. In terms of missing outcome data, the first RCT had a low rate of dropout (<10%), and the survival analysis was performed taking into account the censored data, but no specific information of missed participants could be obtained. The second RCT had a high rate of dropout (approximately 16.8%) and did not include information on missed participants in the final analysis, so there were some concerns about deviations from intended interventions. Overall, the RCTs were of high quality. Although some concerns existed, the iodine status was primarily related to dietary habits rather than additional imposed interventions, so the risk of adverse effect associated with them may have been relatively low and may not be able to affect the study results.

The small sample size and bias of the three observational studies is a concern. The Korean 2015 study did consider the effect of age when analyzing the results and performed subgroup analysis. However, the study did not report any information related to dropout, and the results only included those who completed 1 year of follow-up, so there were some concerns in terms of missing data. The US 1987 and UK 1965 studies did not report any measures related to the control of confounding factors. The former had higher dropping (35%) due to incomplete data, while the latter did not report information related to dropout or no response, so there are some concerns about missing data. In addition, the 1987 USA study used data on estimated iodine intake but did not detail the estimation method and data source. The authors assessed the relationship between estimated iodine intake and year, and the remission rate and year separately to infer the relationship between estimated iodine intake and remission rate, so there were a lot of uncontrollable confounding factors, causing the final bias evaluation of high risk. The UK 1965 study used historical controls, and the baseline PII was significantly inconsistent between the two groups, resulting in a very high risk of bias considering the low comparability between subjects from different years. Overall, the risk of bias was higher in observational studies.

## 4 Discussion

We included two RCTs and three observational studies and concluded that, according to WHO criteria for iodine intake, ATD-treated GD patients had lower rates of recurrence and higher rates of remission when going for adequate iodine intake compared with insufficient or above requirements or excessive iodine intake.

This finding is consistent with the effect of iodine on thyroid autoimmunity. Laboratory studies have shown that the effects of excessive iodine on the thyroid gland include enhanced immunogenicity of thyroglobulin molecules (24) and increased production of reactive oxygen species in thyroid cells (25), which exacerbates local inflammatory responses. In contrast, iodine deficiency has been shown to upregulate the expression of thyroid-specific proteins (26, 27), thereby stimulating thyroid hyperplasia and exacerbating the autoimmune response. Thyroglobulin levels were found to be significantly increased at both low and high iodine intake in infants 6-24 months of age, supporting the laboratory evidence (28).

A review detailed the effect of iodine intake on the synthesizing function of the thyroid gland. Severe iodine deficiency results in a lack of raw materials for thyroid hormones and hypothyroidism. When iodine deficiency is mild to moderate, the thyroid gland’s ability to take up and recycle iodine, and synthesize thyroxine, increases as a substitute for iodine deficiency. With this chronic stimulus, the incidence of nodular toxic goiter and hyperthyroidism increases in the population (29). Epidemiological surveys from Europe also confirm this (30).

These studies on the effect of iodine nutritional status on thyroid synthetic function and thyroid autoimmune response indicating that both low and high iodine may exacerbate the thyroid autoimmune response and affect the thyroid’s synthetic function, which may make the Graves’ disease more difficult to control, or increase the rate of recurrence.

In addition to the five included studies, we identified other studies on iodine status and GD hyperthyroidism during the screening process, which had also been widely cited. However, after carefully checking the full text, we found that among them, some studies aimed to explore the appropriate dose of ATD (31), some aimed to explore the efficacy of iodine agents for GD hyperthyroidism (32, 33), some had too short a follow-up period to assess efficacy or recurrence (34), and some did not involve different levels of iodine intake and did not make statistical extrapolations for the comparison of recurrence rates (35). All of them did not meet the inclusion criteria and study objectives of this review, so we did not include these seemingly relevant studies, but they have contributed to some extent to the interest in this issue.

Apart from UIC, WHO also recommends the thyroid volume size (measured by ultrasound) to assess iodine deficiency (36). Previous studies found that children in iodine-deficient areas had larger thyroid volumes than those in iodine-sufficient areas (37), and goiter due to iodine deficiency persisted after correction of iodine deficiency (38). In addition to iodine intake, ethnicity, genetic factors and environmental factors all influence thyroid volume, which limits the validity of international reference ranges (39). Therefore, surveys should be conducted to determine the normal range of thyroid volume in each region and its correlation with iodine nutritional status.

Limitation: Our ideal study would have set up different iodine nutritional statuses in GD patients on ATD for at least 12 months (18) and observed them 12 months after drug withdrawal. However, very few RCTs met these criteria, so both the duration of ATD treatment and post-withdrawal observation were adjusted to 6 months, and observational studies exploring the effect of iodine status on ATD efficacy were also included. However, this resulted in non-negligible inter-study heterogeneity, preventing us from performing statistical analyses of synthetic effect indicators:

First, the two RCTs involved populations with different iodine nutritional status and had different outcome indicators. Second, among the three observational studies, two studies did not provide UIC and could only estimate the iodine nutritional status; one of the studies did not provide specific numbers of remissions rate and total number of people; the last study involved historical controls and did not provide specific values. Finally, it was also not possible to combine the RCTs and observational studies for quantitative analysis due to differences in study design and quality of evidence. In addition, the studies we included did not provide information on thyroid volume at different iodine intakes, so we were unable to compare the effects of iodine intakes on that issue.

All three of the included observational studies were at some risk of bias, and two studies were older and reported population characteristics that may differ from the current general characteristics of patients. However, in interpreting these studies, we carefully analyzed the actual iodine nutritional status, redefined it according to WHO criteria, and corrected the conclusions of some studies that only mentioned iodine intake and ignored iodine nutritional status. The final conclusions were drawn by combining the results of the five studies, which to some extent compensates for the shortcomings of the individual studies.

In addition to this, when initially performing the literature screening, we planned to include RCTs or observational studies on populations with different etiologies of hyperthyroidism (such as nodular goiter, functional adenomas, etc.). However, during the progress, we found that there are very few studies exploring the effect of iodine nutritional status and they all focus only on the GD, the most common cause of hyperthyroidism. It is very regretful that our article cannot answer the question of the effect of different iodine intake on treatment outcome in recurrence rate for rare causes of hyperthyroidism other than GD. Hopefully, this question will be answered in the future.

Our study is the first as we know to use uniform criteria to evaluate the iodine intake status from different studies, making the results comparable. Available limited evidence may suggest that in the management of patients with GD hyperthyroidism, adequate iodine intake to achieve an appropriate iodine nutritional status may be more helpful in improving the efficacy of ATDs to control thyrotoxicosis and reducing the risk of recurrence.

## Registration and protocol

The review was registered in NIHR PROSPERO, with the original title of “What iodine status should be maintained in patients with GD on ATD therapy” (CRD42022359451). Some changes have occurred in the inclusion and exclusion criteria, which were amended continuously in the process of searching.

## Author contributions

Conceptualization: NT and QX; methodology: QX and XZ; software: QX and XZ; validation: JM and XL; formal analysis: QX; investigation: QX, XZ, and XL; data curation: JM; writing—original draft preparation: QX and XZ; writing—review and editing: NT and YZ; visualization: XL; supervision: NT and YZ; project administration: NT; funding acquisition: NT. All authors contributed to the article and approved the submitted version.

## Supplementary material

Supplementary Material 1 Search strategy.

Supplementary Material 3 The article retrieved and the reason for non-inclusion or exclusion.
