# Supplementary material for: Effect of iodine nutritional status on the recurrence of hyperthyroidism and antithyroid drug efficacy in adult patients with Graves’ disease: a systemic review
Source: Front Endocrinol (Lausanne). 2023 Oct 11;14:1234918. doi: 10.3389/fendo.2023.1234918 (PMC10600371; doi:10.3389/fendo.2023.1234918)
Supplement: Supplementary file 1 [file DataSheet_2.docx]

Supplementary material 2. Assessment of risk of bias.

| **Unique ID** | 1 | **Study ID** | ChiCTR1900028109 | **Assessor** | Peng |
| --- | --- | --- | --- | --- | --- |
| **Ref or Label** | 10.1111/cen.13543 | **Aim** | assignment to intervention (the 'intention-to-treat' effect) | |  |
| **Experimental** | the iodine-supplemented group were given 10 grams of iodized salt every day (about 200 micrograms of iodine) | **Comparator** | the iodine-restricted group were given noniodized salt and a low-iodine or noniodine diet | **Source** | Journal article(s) with results of the trial; Research ethics application |
| **Outcome** | The recurrence rates within 12 months after withdrawal of ATD | **Results** | N/A | **Weight** | 459 |
| **Domain** | **Signalling question** |  |  | **Response** | **Comments** |
| **Bias arising from the randomization process** | 1.1 Was the allocation sequence random? | |  | Y | patients (n = 459) were randomly assigned to iodine-supplemented and iodine-restricted groups. |
|  | 1.2 Was the allocation sequence concealed until participants were enrolled and assigned to interventions? | | | PY |  |
|  | 1.3 Did baseline differences between intervention groups suggest a problem with the randomization process? | | | N | The 2 groups were similar in age, gender, thyroid function, UIC and TRAb level before Intervention. At baseline, the UIC of the groups was 60 (44-81) μg/L vs 58 (42-87) μg/L; thus, all these patients were categorized as iodine-restricted status according to WHO/UNICEF/ICCIDD (UIC less than 100 μg/L), and there was no significant difference between the groups. |
|  | **Risk of bias judgement** |  |  | **Low** |  |
| **Bias due to deviations from intended interventions** | 2.1.Were participants aware of their assigned intervention during the trial? | | | PY | Patients the in iodine-supplemented group were given 10 grams of iodized salt every day (about 200 micrograms of iodine), while the iodine-restricted group were given noniodized salt and a low-iodine or noniodine diet. The change in dietary seems to be obvious. |
|  | 2.2.Were carers and people delivering the interventions aware of participants' assigned intervention during the trial? | | | PY |  |
|  | 2.3. If Y/PY/NI to 2.1 or 2.2: Were there deviations from the intended intervention that arose because of the experimental context? | | | PY | They repoted 7 patients in iodine supplemented group and 5 patients in iodine restricted group changed their mind after randomization. |
|  | 2.4 If Y/PY to 2.3: Were these deviations likely to have affected the outcome? | | | PN | The deviations only accounted for little part in the total participants. |
|  | 2.5. If Y/PY/NI to 2.4: Were these deviations from intended intervention balanced between groups? | | | NA |  |
|  | 2.6 Was an appropriate analysis used to estimate the effect of assignment to intervention? | | | PY | A Kaplan-Meier log rank survival and a Cox regression model analysis were performed to compare recurrence rate between 2 groups. So they included the censored data. |
|  | 2.7 If N/PN/NI to 2.6: Was there potential for a substantial impact (on the result) of the failure to analyse participants in the group to which they were randomized? | | | NA |  |
|  | **Risk of bias judgement** |  |  | **Some concerns** |  |
| **Bias due to missing outcome data** | 3.1 Were data for this outcome available for all, or nearly all, participants randomized? | | | Y | The number of participants with missing outcome is sufficiently small. |
|  | 3.2 If N/PN/NI to 3.1: Is there evidence that result was not biased by missing outcome data? | | | NA |  |
|  | 3.3 If N/PN to 3.2: Could missingness in the outcome depend on its true value? | | | NA |  |
|  | 3.4 If Y/PY/NI to 3.3: Is it likely that missingness in the outcome depended on its true value? | | | NA |  |
|  | **Risk of bias judgement** |  |  | **Low** |  |
| **Bias in measurement of the outcome** | 4.1 Was the method of measuring the outcome inappropriate? | |  | N | The methods of clinical examination for thyroid enlargement, assessment of thyroid function , TRAb and urinary iodine were presented in detail and pre-specified. |
|  | 4.2 Could measurement or ascertainment of the outcome have differed between intervention groups? | | | N | The measuremennts were applied to all participants in two groups. |
|  | 4.3 Were outcome assessors aware of the intervention received by study participants? | | | PY | Nobody seems to be blinded in this trial. |
|  | 4.4 If Y/PY/NI to 4.3: Could assessment of the outcome have been influenced by knowledge of intervention received? | | | PN | The measurement of the outcome is object with strict standard. |
|  | 4.5 If Y/PY/NI to 4.4: Is it likely that assessment of the outcome was influenced by knowledge of intervention received? | | | NA |  |
|  | **Risk of bias judgement** |  |  | **Low** |  |
| **Bias in selection of the reported result** | 5.1 Were the data that produced this result analysed in accordance with a pre-specified analysis plan that was finalized before unblinded outcome data were available for analysis? | | | Y | A Mann-Whitney and Wilcoxon signed rank test were used for comparing clinical characteristics, UIC, TRAb and thyroid function between the iodine-supplemented and iodine-restricted groups. A Kaplan-Meier log rank survival and a Cox regression model analysis were performed to compare recurrence rate between 2 groups. Data are expressed as n (%) or means ± SD or medians (interquartile range). P < .05 was considered statistically significant. |
|  | 5.2 ... multiple eligible outcome measurements (e.g. scales, definitions, time points) within the outcome domain? | | | PN | There is only one possible way in which the outcome domain can be analyzed. |
|  | 5.3 ... multiple eligible analyses of the data? | |  | PN | There is only one possible way in which the outcome domain can be analyzed. |
|  | **Risk of bias judgement** |  |  | **Low** |  |
| **Overall bias** | **Risk of bias judgement** |  |  | **Some concerns** | Nobody seems to be blinded in this trial. However, the measurements were objective with strict standard. Thus the influence is unpredictable. |
|  |  |  |  |  |  |
|  |  |  |  |  |  |
| **Unique ID** | 2 | **Study ID** | inaccessible | **Assessor** | Peng |
| **Ref or Label** | 16834930 | **Aim** | assignment to intervention (the 'intention-to-treat' effect) | |  |
| **Experimental** | (group A) were given non-iodinated salt | **Comparator** | (group B) were given USI [(35±15) mg iodide/kg] | **Source** | Journal article(s) with results of the trial |
| **Outcome** | UIC, thyroid hormones, PTU doses and positive rate of TRAb | **Results** | N/A | **Weight** | 124 |
| **Domain** | **Signalling question** |  |  | **Response** | **Comments** |
| **Bias arising from the randomization process** | 1.1 Was the allocation sequence random? | |  | Y | One hundred and twenty-four patients with untreated Graves' disease were recruited at the thyroid section of the Endocrinology Department of PUMCH from January 2001 to August 2002, and were randomized into two groups. |
|  | 1.2 Was the allocation sequence concealed until participants were enrolled and assigned to interventions? | | | PY |  |
|  | 1.3 Did baseline differences between intervention groups suggest a problem with the randomization process? | | | N | The age and gender distributions，the body weight, and the degree of goiter of the two groups were not statistically different |
|  | **Risk of bias judgement** |  |  | **Low** |  |
| **Bias due to deviations from intended interventions** | 2.1.Were participants aware of their assigned intervention during the trial? | | | PY | Nobody in this trail seems to be blinded. |
|  | 2.2.Were carers and people delivering the interventions aware of participants' assigned intervention during the trial? | | | PY |  |
|  | 2.3. If Y/PY/NI to 2.1 or 2.2: Were there deviations from the intended intervention that arose because of the experimental context? | | | PY | In group A, 3 patients were transferred from propylthiouracil (PTU) to radioiodine therapy because of severe granulocytopenia, rash and gastrointestinal reaction. Two patients in group A withdrew from the study. Five patients from group B were lost to follow-up. In group B, 3 patients were given radioiodine therapy instead of PTU because of severe rash or granulocytopenia. Ten patients in group B were lost to follow-up. |
|  | 2.4 If Y/PY to 2.3: Were these deviations likely to have affected the outcome? | | | PY | The rate for both groups was 16.8 %, which is relatively high. |
|  | 2.5. If Y/PY/NI to 2.4: Were these deviations from intended intervention balanced between groups? | | | Y | The rate of loss of follow-up was 15.6 % (7/45) for group A and 17.9 % (10/56) for group B. |
|  | 2.6 Was an appropriate analysis used to estimate the effect of assignment to intervention? | | | PN | The data only included the participants adhering to the protocol. |
|  | 2.7 If N/PN/NI to 2.6: Was there potential for a substantial impact (on the result) of the failure to analyse participants in the group to which they were randomized? | | | PN | In theory, the intervention could not bring series adverse effect to the participants. Thus, the loss of follow-up may neither directly relate to the intervention, nor influence the result. |
|  | **Risk of bias judgement** |  |  | **Some concerns** |  |
| **Bias due to missing outcome data** | 3.1 Were data for this outcome available for all, or nearly all, participants randomized? | | | PN | Forty-five patients out of 55 patients in group A and 56 patients out of 69 patients in group B were followed up for 6 months. |
|  | 3.2 If N/PN/NI to 3.1: Is there evidence that result was not biased by missing outcome data? | | | N | There was neither bias correction nor sensitivity analysis. |
|  | 3.3 If N/PN to 3.2: Could missingness in the outcome depend on its true value? | | | PN | In theory, the intervention could not bring series adverse effect to the participants. Thus, the loss of follow-up may neither directly relate to the intervention, nor influence the result. |
|  | 3.4 If Y/PY/NI to 3.3: Is it likely that missingness in the outcome depended on its true value? | | | NA |  |
|  | **Risk of bias judgement** |  |  | **Low** |  |
| **Bias in measurement of the outcome** | 4.1 Was the method of measuring the outcome inappropriate? | |  | N | The measurements of urinary iodide, thyroid hormones, PTU doses and positive rate of TRAb were presented in detail with strict standard. |
|  | 4.2 Could measurement or ascertainment of the outcome have differed between intervention groups? | | | N | The measurements were applied to all patients in both group. |
|  | 4.3 Were outcome assessors aware of the intervention received by study participants? | | | PY | Nobody seems to be blinded in the trial. |
|  | 4.4 If Y/PY/NI to 4.3: Could assessment of the outcome have been influenced by knowledge of intervention received? | | | PN | The measurements were objective with strict standard. |
|  | 4.5 If Y/PY/NI to 4.4: Is it likely that assessment of the outcome was influenced by knowledge of intervention received? | | | NA |  |
|  | **Risk of bias judgement** |  |  | **Low** |  |
| **Bias in selection of the reported result** | 5.1 Were the data that produced this result analysed in accordance with a pre-specified analysis plan that was finalized before unblinded outcome data were available for analysis? | | | Y | Statistical analysis was made by paired and/or unpaired Student's t test to compare age, body weight and the levels of serum TT4, TT3, FT4, and FT3. Fisher's exact test was employed to evaluate the degree of goiter, PTU dose, and sex ratio in groups A and B. Urinary iodine was compared by nonparametric statistics. |
|  | 5.2 ... multiple eligible outcome measurements (e.g. scales, definitions, time points) within the outcome domain? | | | PN | There is only one possible way in which the outcome domain can be measured. |
|  | 5.3 ... multiple eligible analyses of the data? | |  | PN | There is only one possible way in which the outcome domain can be measured. |
|  | **Risk of bias judgement** |  |  | **Low** |  |
| **Overall bias** | **Risk of bias judgement** |  |  | **Some concerns** | The loss of follow-up is relatively high (16.8% on average), and nobody seems to be blinded in this trial. However, In theory, the intervention could not bring series adverse effect to the participants. Thus, the loss of follow-up may neither directly relate to the intervention, nor influence the result. The measurements were objective with strict standard, so they make the bias unpredictable. |

Study: 2015, Korean, Park, S. M., *et al*.

| Planning stage | P1. List the important confounding factors relevant to all or most studies on this topic. Specify whether these are particular to specific exposures-outcome combinations. | The age, the course and severity of graves' disease, the time of ATD treatment |
| --- | --- | --- |
|  | P2. Will the review use the ROBINS-E assessment of appropriateness (important aspects of “study sensitivity”)? | No |
| Preliminary Considerations | A1. Specify the numerical result being assessed. | The rate of relapse |
|  | B1. Did the authors make any attempt to control for confounding? | N |
|  | B2. **If N/PN to B1:** Is there sufficient potential for confounding that an unadjusted result should not be considered further? | PN |
|  | B3. Was the method of measuring exposure inappropriate? | N |
|  | B4. Was the method of measuring the outcome inappropriate? | N |
|  | C1. Specify the outcome to which this result relates | UIC, UIC/Ucr, FT4, TSH |
|  | C2. Specify the participant group on which this result was based. | Patients who had taken ATD for at least 12 months and then stopped their ATD |
|  | C3. What is the exposure being measured and how was it measured or assessed? | Iodine status measured by UIC |
|  | C4. Was exposure analysed as a quantitative (rather than a categorical) variable? | Y |
|  | C5. Did repeated measurements of exposure over time (for each participant) contribute to the analysis that produced this result? | Y |
|  | C6. **If Y/PY to C5,** was a single estimate of each participant’s exposure level derived from the repeated measurements of exposure over time? | PY |
|  | C10. Specify the relationship analysed to produce this result. For example, this may be a quadratic relationship of cumulative exposure with the log odds of the outcome, or a risk ratio for the outcome comparing exposed with unexposed individuals. | Compare the remission rate and relapse rate between groups |
|  | D1. Specify the population of interest | We included the patients who had taken ATD for at least 12 months and then stopped their ATD based on the following criteria: normalization of increased T3 and free T4, restoration of suppressed TSH, and increased anti-TSH receptor antibody (TRAb). Patients who were followed-up more than 12 months after therapy cessation were finally included |
|  | D2. Specify the exposure | Iodine status |
|  | D3. Specify the exposure window | Patients stopped their ATD between October 2011 and April 2013 |
|  | D4. Specify how exposure over time should be summarized | Average iodine status measured by UIC |
|  | E. Evaluation of confounding factors | The age (measured by age; controlled in the analysis) |
|  |  | The severity of graves' disease (measured by FT4, TSH, and TRAb; controlled in the analysis) |
|  |  | The time of ATD treatment (measured by year; controlled in the analysis) |
| Domain 1: Risk of bias due to confounding (Variant A) | 1.1 Did the authors control for all the important confounding factors for which this was necessary? | PY  The inclusion criteria limited the function status of thyroid and ATD treatment course, and subgroup analysis controlled the age. However, there may be unmeasured residual confounding |
|  | 1.2 Were confounding factors that were controlled for (and for which control was necessary) measured validly and reliably by the variables available in this study? | Y  The measuments of the confounding factors are objective with strict standard. |
|  | 1.3 Did the authors control for any variables after the start of the exposure period being studied that could have been affected by the exposure? | PN  No control seems to happen after the start of the exposure. |
|  | 1.4 Did the use of negative controls, or other considerations, suggest serious uncontrolled confounding? | N  There isn't negative control in the trial. |
|  | Risk of bias (due to confounding) in the estimated effect of exposure on the outcome | Low Risk of Bias, except for concerns about residual confounding |
| Domain 2: Risk of bias arising from measurement of the exposure (Variant B) | 2.1 Does the measured exposure well-characterize the exposure metric specified to be of interest in this study? *[This was specified in the answers to D2, D3 and D4]* | Y  The intraday coefficient of variation for UIC ranged from 0.3 to 1.2% and the interday coefficient of variation ranged from 1.4 to 3.3%. |
|  | 2.2 Was the exposure likely to be measured with error, or misclassified? | PN  Urinary creatinine was measured using the Cobas Integra 800 instrument (Roche Diagnostics, Basel, Switzerland) |
|  | Risk of bias (arising from measurement of exposure) in the estimated effect of exposure on the outcome: | Low Risk of Bias |
| Domain 3: Risk of bias in selection of participants into the study (or into the analysis) | 3.1  Did follow-up begin at (or close to) the start of the exposure window for most participants? *[The exposure window is specified in D3]* | PY  The exposure window started just after the withdrwal of ATD. |
|  | 3.3 Was selection of participants into the study (or into the analysis) based on participant characteristics observed after the start of the exposure window being studied? *[The exposure window is specified in D3]* | N  The measurements were implemented after the enrollment of participants |
|  | Risk of bias (due to selection of participants into the study) in the estimated effect of exposure on the outcome: | Low Risk of Bias |
| Domain 4: Risk of bias due to post-exposure interventions | 4.1 Were there post-exposure interventions that were influenced by prior exposure during the follow-up period? | PN  All participants in both groups received the measurements, with no additional intervention. |
|  | Risk of bias (due post-exposure interventions) in the estimated effect of exposure on the outcome: | Low Risk of Bias |
| Domain 5: Risk of bias due to missing data | 5.1 Were complete data on exposure status, confounding variables and the outcome available for all, or nearly all, participants? | NI  The article didn't provide information about missing data |
|  | 5.2 Were complete data on the outcome available for all, or nearly all, participants? |  |
|  | 5.3 Were complete data on confounding variables available for all, or nearly all, participants? |  |
|  | 5.4 Is the result based on a complete case analysis? | Y  Only those were followed-up more than 12 months after therapy cessation were finally included |
|  | 5.5 Was exclusion from the analysis because of missing data (in exposure, confounders or the outcome) likely to be related to the true value of the outcome? | PN  The iodine status relates to dietary habits, which requires no additional intervention and has little relation to their response or missing |
|  | 5.6 Were all or most predictors of missingness (in exposure, confounders or the outcome) included in the analysis model? | WY (Yes, mostly or probably)  The results analysis only adjust according to age |
|  | Risk of bias (due to selection of participants into the study) in the estimated effect of exposure on the outcome: | Some Concerns |
| Domain 6: Risk of bias arising from measurement of outcomes | 6.1 Could measurement or ascertainment of the outcome have differed between exposure groups or levels of exposure? | N  All participants in both group received the same measurements of UIC and thyroid hormone |
|  | 6.2 Were outcome assessors aware of study participants’ exposure history? | PY  Nobody seems to be blinded in the trial |
|  | 6.3 Could assessment of the outcome have been influenced by knowledge of participants’ exposure history? | PN  The measurements are objective with strict standard |
|  | Risk of bias (arising from measurement of outcomes) in the estimated effect of exposure on the outcome: | Low Risk of Bias |
| Dom ain 7: Risk of bias in selection of the reported result | 7.1 Was the result reported in accordance with an available, pre-determined analysis plan? | Y  A Mann-Whitney test and t test were used for comparing clinical characteristics between the remission and relapse groups. A Kruskal-Wallis test and ANOVA were used for comparisons among the four groups. An exact χ2 test was performed to compare remission and relapse rates between excessive and average iodine intake group. Subgroup analysis for the age groups was performed by the Kruskal-Wallis test. Bonferroni's correction was applied to the post hoc analysis of the between-age group comparisons to allow for the number of comparisons performed. p < 0.05 was considered statistically significant |
|  | Risk of bias (due to selection of the reported result) in the estimated effect of exposure on the outcome: | Low Risk of Bias |
| Overall risk of bias | Overall Risk-of-Bias Rating: | **Some Concerns**  They controlled several important counfounding factors such as age, but lack information about missing data and residual counfounding |
|  | What is the predicted direction of bias? | Insufficient information available |
|  | Is the overall risk of bias sufficiently high, in the context of its likely direction and the magnitude of the estimated exposure effect, to threaten conclusions about whether the exposure has an important effect on the outcome? | No |

Study: 1987, USA, Solomon, B. L., *et al*.

| Planning stage | P1. List the important confounding factors relevant to all or most studies on this topic. Specify whether these are particular to specific exposures-outcome combinations. | The age, the course and severity of graves' disease, the time of ATD treatment |
| --- | --- | --- |
|  | P2. Will the review use the ROBINS-E assessment of appropriateness (important aspects of “study sensitivity”)? | No |
| Preliminary Considerations | A1. Specify the numerical result being assessed. | The percent or remission |
|  | B1. Did the authors make any attempt to control for confounding? | N |
|  | B2. **If N/PN to B1:** Is there sufficient potential for confounding that an unadjusted result should not be considered further? | PN |
|  | B3. Was the method of measuring exposure inappropriate? | PN |
|  | B4. Was the method of measuring the outcome inappropriate? | N |
|  | C1. Specify the outcome to which this result relates | FT4, TSH |
|  | C2. Specify the participant group on which this result was based. | Patients with an established diagnosis of Graves’ disease |
|  | C3. What is the exposure being measured and how was it measured or assessed? | Iodine status measured by estimated iodine intake |
|  | C4. Was exposure analysed as a quantitative (rather than a categorical) variable? | Y |
|  | C5. Did repeated measurements of exposure over time (for each participant) contribute to the analysis that produced this result? | Y |
|  | C6. **If Y/PY to C5,** was a single estimate of each participant’s exposure level derived from the repeated measurements of exposure over time? | PN |
|  | C7. **If N/PN to C6,** was the analysis based on splitting participants’ follow up time according to exposure status and/or magnitude? | PN |
|  | C9. **If N/PN to C7,** how were repeat measurements used? | The use of repeated exposure measurements to characterize changes in exposure lever over time |
|  | C10. Specify the relationship analysed to produce this result. For example, this may be a quadratic relationship of cumulative exposure with the log odds of the outcome, or a risk ratio for the outcome comparing exposed with unexposed individuals. | Analyze the relation between remission rate and estimated iodine intake |
|  | D1. Specify the population of interest | The medical records of all patients with an established diagnosis of Graves disease seen in our thyroid clinic between July 1973 and January 1986 were reviewed. Excluded from the study were patients treated with a short course (6 months or less) of antithyroid drugs in preparation for surgery or 131I therapy; with euthyroid Graves disease; continuing on antithyroid drug therapy at the time of review; and referred for definitive therapy, but who had been treated at another facility. |
|  | D2. Specify the exposure | Iodine status |
|  | D3. Specify the exposure window | 1973-1985 |
|  | D4. Specify how exposure over time should be summarized | Average iodine status measured by estimated iodine intake |
|  | E. Evaluation of confounding factors | The age (measured by age; not controlled in the analysis; measured validly and reliably by this variable; without evidence that controlling for it was unnecessary) |
|  |  | The severity of graves' disease (measured by FT4, TSH, and TRAb; controlled in the analysis) |
|  |  | The time of ATD treatment (measured by year; controlled in the analysis) |
| Domain 1: Risk of bias due to confounding (Variant A) | 1.1 Did the authors control for all the important confounding factors for which this was necessary? | SN (no, and uncontrolled confounding was probably substantial)  They didn't take control of some important factors, and lack the baseline characteristic |
|  | Risk of bias (due to confounding) in the estimated effect of exposure on the outcome | High Risk of Bias |
| Domain 2: Risk of bias arising from measurement of the exposure (Variant C) | 2.1 Does the measured exposure well-characterize the exposure metric specified to be of interest in this study? *[This was specified in the answers to D2, D3 and D4]* | PY  They didn't describe the method use to estimate the iodine intake in detail. But the data seems to come from national database |
|  | 2.2 Was the exposure likely to be measured with error, or misclassified? | WY (yes, but probably not a substantial amount)  The data seems to come from national database |
|  | 2.3 Could mismeasurement or misclassification of exposure have been differential (i.e. related to the outcome or risk of the outcome)? | PN  The exposure data seems to be obtained from sources external to the study |
|  | 2.4 Is non-differential measurement error likely to bias the estimated effect of exposure on outcome? | PN  The exposure data from sources external to the study, thus having little relation with the result |
|  | Risk of bias (arising from measurement of exposure) in the estimated effect of exposure on the outcome: | Low Risk of Bias |
| Domain 3: Risk of bias in selection of participants into the study (or into the analysis) | 3.1  Did follow-up begin at (or close to) the start of the exposure window for most participants? *[The exposure window is specified in D3]* | PY  The exposure window started just after the withdrwal of ATD. |
|  | 3.3 Was selection of participants into the study (or into the analysis) based on participant characteristics observed after the start of the exposure window being studied? *[The exposure window is specified in D3]* | N  The measurements were implemented after the enrollment of participants |
|  | Risk of bias (due to selection of participants into the study) in the estimated effect of exposure on the outcome: | Low Risk of Bias |
| Domain 4: Risk of bias due to post-exposure interventions | 4.1 Were there post-exposure interventions that were influenced by prior exposure during the follow-up period? | PN  All participants in both groups received the measurements, with no additional intervention. |
|  | Risk of bias (due post-exposure interventions) in the estimated effect of exposure on the outcome: | Low Risk of Bias |
| Domain 5: Risk of bias due to missing data | 5.1 Were complete data on exposure status, confounding variables and the outcome available for all, or nearly all, participants? | N  106 patients met eligibility requirements for inclusion in the study. Incomplete data necessitated those 37 patients be dropped from the final analysis. |
|  | 5.2 Were complete data on the outcome available for all, or nearly all, participants? | N  The article didn't provide information about missing data |
|  | 5.3 Were complete data on confounding variables available for all, or nearly all, participants? |  |
|  | 5.4 Is the result based on a complete case analysis? | Y  Only those were followed-up more than 6 months after therapy cessation were finally included |
|  | 5.5 Was exclusion from the analysis because of missing data (in exposure, confounders or the outcome) likely to be related to the true value of the outcome? | PN  The iodine status relates to dietary habits, which requires no additional intervention and has little relation to their response or missing |
|  | 5.6 Were all or most predictors of missingness (in exposure, confounders or the outcome) included in the analysis model? | PN  The results anaylsis didn't make any adjustment to the counfounding factor |
|  | Risk of bias (due to selection of participants into the study) in the estimated effect of exposure on the outcome: | High Risk of Bias |
| Domain 6: Risk of bias arising from measurement of outcomes | 6.1 Could measurement or ascertainment of the outcome have differed between exposure groups or levels of exposure? | N  All paticipants in both groups received the same measurements of thyroid hormone, and the data about exposure seems to be obtained from other sources |
|  | 6.2 Were outcome assessors aware of study participants’ exposure history? | PY  Nobody seems to be blinded in the trial |
|  | 6.3 Could assessment of the outcome have been influenced by knowledge of participants’ exposure history? | PN  The measurements are objective with strict standard |
|  | Risk of bias (arising from measurement of outcomes) in the estimated effect of exposure on the outcome: | Low Risk of Bias |
| Domain 7: Risk of bias in selection of the reported result | 7.1 Was the result reported in accordance with an available, pre-determined analysis plan? | PY  The relationship between estimated daily iodine intake (11-13) and remission rates observed in the present study from 1973 to 1985 is shown in figure |
|  | Risk of bias (due to selection of the reported result) in the estimated effect of exposure on the outcome: | Low Risk of Bias |
| Overall risk of bias | Overall Risk-of-Bias Rating: | **High Risk of Bias**  They didn't control any counfounding factors through data analysis, and lack the baseline characteristic. What's worse, they didn't present the method to estimate the iodine status in detail, which makes it hard to tell their validity |
|  | What is the predicted direction of bias? | Insufficient information available |
|  | Is the overall risk of bias sufficiently high, in the context of its likely direction and the magnitude of the estimated exposure effect, to threaten conclusions about whether the exposure has an important effect on the outcome? | Can't tell |

Study: 1965, Britain, Alexander, W. D., *et al*.

| Planning stage | P1. List the important confounding factors relevant to all or most studies on this topic. Specify whether these are particular to specific exposures-outcome combinations. | The age, the course and severity of graves' disease, the time of ATD treatment |
| --- | --- | --- |
|  | P2. Will the review use the ROBINS-E assessment of appropriateness (important aspects of “study sensitivity”)? | No |
| Preliminary Considerations | A1. Specify the numerical result being assessed. | The rate of relapse |
|  | B1. Did the authors make any attempt to control for confounding? | N |
|  | B2. **If N/PN to B1:** Is there sufficient potential for confounding that an unadjusted result should not be considered further? | PN |
|  | B3. Was the method of measuring exposure inappropriate? | N |
|  | B4. Was the method of measuring the outcome inappropriate? | N |
|  | C1. Specify the outcome to which this result relates | PII, FT4, TSH |
|  | C2. Specify the participant group on which this result was based. | Patients  had had thyrotoxicosis treated with carbimazole or methylthiouracil for 1 to 2 years were investigated |
|  | C3. What is the exposure being measured and how was it measured or assessed? | Iodine status measured by PII |
|  | C4. Was exposure analysed as a quantitative (rather than a categorical) variable? | Y |
|  | C5. Did repeated measurements of exposure over time (for each participant) contribute to the analysis that produced this result? | N |
|  | C10. Specify the relationship analysed to produce this result. For example, this may be a quadratic relationship of cumulative exposure with the log odds of the outcome, or a risk ratio for the outcome comparing exposed with unexposed individuals. | Compare the relapse rate between groups |
|  | D1. Specify the population of interest | Patients had had thyrotoxicosis treated with carbimazole or methylthiouracil for 1 to 2 years. |
|  | D2. Specify the exposure | Iodine status |
|  | D3. Specify the exposure window | Patients stopped their ATD before the trial |
|  | D4. Specify how exposure over time should be summarized | Average iodine status measured by PII |
|  | E. Evaluation of confounding factors | The age (measured by age; not controlled in the analysis; measured validly and reliably by this variable; without evidence that controlling for it was unnecessary) |
|  |  | The severity of graves' disease (measured by FT4, TSH, and TRAb; controlled in the analysis) |
|  |  | The time of ATD treatment (measured by year; controlled in the analysis) |
| Domain 1: Risk of bias due to confounding (Variant A) | 1.1 Did the authors control for all the important confounding factors for which this was necessary? | SN (no, and uncontrolled confounding was probably substantial)  They didn't take control of some important factors, and lack the baseline characteristic |
|  | Risk of bias (due to confounding) in the estimated effect of exposure on the outcome | High Risk of Bias |
| Domain 2: Risk of bias arising from measurement of the exposure (Variant A) | 2.1 Does the measured exposure well-characterize the exposure metric specified to be of interest in this study? *[This was specified in the answers to D2, D3 and D4]* | Y  The iodine status was measured by plasma-inorganic-iodine |
|  | 2.2 Was the exposure likely to be measured with error, or misclassified? | N  The exposure was consistently assessed using established or validated methods that measure the exposure directly |
|  | Risk of bias (arising from measurement of exposure) in the estimated effect of exposure on the outcome: | Low Risk of Bias |
| Domain 3: Risk of bias in selection of participants into the study (or into the analysis) | 3.1  Did follow-up begin at (or close to) the start of the exposure window for most participants? *[The exposure window is specified in D3]* | PY  The exposure window started just after the withdrwal of ATD. |
|  | 3.3 Was selection of participants into the study (or into the analysis) based on participant characteristics observed after the start of the exposure window being studied? *[The exposure window is specified in D3]* | N  The measurements were implemented after the enrollment of participants |
|  | Risk of bias (due to selection of participants into the study) in the estimated effect of exposure on the outcome: | Low Risk of Bias |
| Domain 4: Risk of bias due to post-exposure interventions | 4.1 Were there post-exposure interventions that were influenced by prior exposure during the follow-up period? | PN  All participants in both groups received the measurements, with no additional intervention |
|  | Risk of bias (due post-exposure interventions) in the estimated effect of exposure on the outcome: | Low Risk of Bias |
| Domain 5: Risk of bias due to missing data | 5.1 Were complete data on exposure status, confounding variables and the outcome available for all, or nearly all, participants? | NI  The article didn't provide information about missing data |
|  | 5.2 Were complete data on the outcome available for all, or nearly all, participants? |  |
|  | 5.3 Were complete data on confounding variables available for all, or nearly all, participants? |  |
|  | 5.4 Is the result based on a complete case analysis? | PY  The article only reported the data of complete case |
|  | 5.5 Was exclusion from the analysis because of missing data (in exposure, confounders or the outcome) likely to be related to the true value of the outcome? | PN  The iodine status relates to dietary habits, which requires no additional intervention and has little relation to their response or missing |
|  | 5.6 Were all or most predictors of missingness (in exposure, confounders or the outcome) included in the analysis model? | PN  They didn't control any confounding factors through data analysis |
|  | Risk of bias (due to selection of participants into the study) in the estimated effect of exposure on the outcome: | High Risk of Bias |
| Domain 6: Risk of bias arising from measurement of outcomes  Domain 7: Risk of bias in selection of the reported result | 6.1 Could measurement or ascertainment of the outcome have differed between exposure groups or levels of exposure? | PN  All participants in both groups received the same measurements of UIC and thyroid hormone |
|  | 6.2 Were outcome assessors aware of study participants’ exposure history? | PY  Nobody seems to be blinded in the trial |
|  | 6.3 Could assessment of the outcome have been influenced by knowledge of participants’ exposure history? | PN  The measurements are objective with strict standard |
|  | Risk of bias (arising from measurement of outcomes) in the estimated effect of exposure on the outcome: | Low Risk of Bias |
|  | 7.1 Was the result reported in accordance with an available, pre-determined analysis plan? | Y  A chi-square test was used for comparing the relapse rate |
|  | Risk of bias (due to selection of the reported result) in the estimated effect of exposure on the outcome: | Low Risk of Bias |
| Overall risk of bias | Overall Risk-of-Bias Rating: | **Very High Risk of Bias**  They didn't control any counfounding factors through data analysis. What's worse, they compared the relapse rate of participants in the present trial with that of participants in the previous trial, leading to many factors out of control |
|  | What is the predicted direction of bias? | Insufficient information available |
|  | Is the overall risk of bias sufficiently high, in the context of its likely direction and the magnitude of the estimated exposure effect, to threaten conclusions about whether the exposure has an important effect on the outcome? | No |
