# Supplementary material for: Effect of iodine nutritional status on the recurrence of hyperthyroidism and antithyroid drug efficacy in adult patients with Graves’ disease: a systemic review
Source: Front Endocrinol (Lausanne). 2023 Oct 11;14:1234918. doi: 10.3389/fendo.2023.1234918 (PMC10600371; doi:10.3389/fendo.2023.1234918)
Supplement: Supplementary Material 1 — Search strategy. [file DataSheet_1.docx]

***The Cochrane Library***

#1 [mh "Graves disease"]

#2 (grave* near/7 (diseas* or thyrotoxicos* or hyperthyr*)):ti,ab,kw

#3 (basedow* near/7 (diseas* or syndrom*)):ti,ab,kw

#4 (#1 or #2 or #3)

#5 [mh Iodine]

#6 [mh Iodides]

#7 (Iodine in All Text or Iodize* in All Text or “dietary iodine” in All Text or “iodine intake” in All Text)

#8 (#5 or #6 or #7)

#9 [mh "Antithyroid Agents"]

#10 ((antithyroid* or anti-thyroid*) near/7 (therap* or treatment* or agent* or drug* or substanc* or compound*)):ti,ab,kw

#11 (carbimazole* or methimazole* or methylthiouracil* or propylthiouracil* or thiouracil*):ti,ab,kw

#12 [mh Carbimazole]

#13 [mh Methimazole]

#14 [mh Methylthiouracil]

#15 [mh Propylthiouracil]

#16 [mh Thiouracil]

#17 {or #9-#16}

#18 (#4 and #8 and #17)

#19 (#18 not radio*)

**MEDLINE (Ovid SP)**

1 exp Graves Disease/

2 (grave* adj6 (diseas* or thyrotoxicos* or hyperthyr*)).tw,ot.

3 (basedow* adj6 (diseas* or syndrom*)).tw,ot.

4 or/1-3

5 exp Iodine/

6 exp Iodides/

7 (Iodine or Iodize* or dietary iodine or iodine intake).tw,ot.

8 or/5-7 not radio* .mp.

9. exp Antithyroid Agents/

10. ((antithyroid* or anti-thyroid*) adj6 (therap* or treatment* or agent* or drug* or substanc* or compound*)).tw,ot.

11. (carbimazole* or methimazole* or methylthiouracil* or propylthiouracil* or thiouracil*).tw,ot.

12. exp Carbimazole/

13. exp Methimazole/

14. exp Methylthiouracil/

15. exp Propylthiouracil/

16. exp Thiouracil/

17. or/9-16

[18-28: Cochrane RCT Filter – sensitivity maximizing version 2008]

18. randomized controlled trial.pt.

19. controlled clinical trial.pt.

20. randomized.ab.

21. placebo.ab.

22. drug therapy.fs.

23. randomly.ab.

24. trial.ab.

25. groups.ab.

26. or/18-25

27. exp animals/ not humans/

28. 26 not 27

29. 4 and 8 and 17 and 28

**EMBASE** **(Ovid SP)**

1 exp Graves disease/

2 (basedow adj6 (diseas* or syndrom*)).tw.

3 (grave* adj6 (disease* or hyperthyr* or thyrotoxicos*)).tw.

4 or/1-3

5 exp Iodine/

6 exp Iodides/

7 (Iodine or Iodize* or dietary iodine or iodine intake).tw,ot.

8 or/5-7 not radio* .mp.

9. exp antithyroid agent/

10. ((antithyroid* or anti-thyroid*) adj6 (therap* or treatment* or agent* or drug* or substanc* or compound*)).tw.

11. exp carbimazole/

12. exp thiamazole/

13. exp methylthiouracil/

14. exp propylthiouracil/

15. exp thiouracil/

16. (carbimazole* or methimazole* or methylthiouracil* or propylthiouracil* or thiouracil*).tw.

17. or/9-16

18. 4 and 8 and 17

[19: Wong 2006 – small drop in sensitivity, substantive gain in specificity filter]

19. random*.tw. or clinical trial*.mp. or exp treatment outcome/

20. 18 and 19

21. limit 20 to embase
